# Prognostic significance of tumor markers in T4a gastric cancer

**DOI:** 10.1186/1477-7819-10-68

**Published:** 2012-04-27

**Authors:** Xiaowen Liu, Hong Cai, Yanong Wang

**Affiliations:** 1 Department of Abdominal Surgery, Cancer Hospital, Fudan University, 270 Dong An Road, Shanghai, 200032, People’s Republic of China; 2 Department of Oncology, Shanghai Medical College, Fudan University, Shanghai, People’s Republic of China

**Keywords:** Tumor markers, Gastric cancer, Prognosis

## Abstract

**Background:**

The clinical importance of preoperative tumor markers remain elusive in gastric cancer. The aim of this study was to evaluate the prognostic value of AFP, CEA, CA19-9, and CA50 in T4a stage gastric cancer.

**Methods:**

Two hundred and seventy-three T4a gastric cancer patients who underwent curative D2 gastrectomy between 1996 and 2005 were evaluated. The correlation between tumor markers and clinicopathologic characteristics and prognostic value of preoperative tumor markers were investigated.

**Results:**

Correlation analysis showed that AFP was associated with Borrmann type (*P* = 0.010); CEA with sex (*P* = 0.029), tumors site (*P* = 0.014), and N stage (*P* = 0.001); CA19-9 with age (*P* = 0.047), tumor site (*P* = 0.011), lymphovascular invasion (*P* = 0.004), and N stage (*P* = 0.000); CA50 with age (*P* = 0.017), tumor site (*P* = 0.004), tumor size (*P* = 0.014), and N stage (*P* = 0.000). Multivariate analysis showed that the positivity of preoperative CEA, CA19-9, and CA50 were major independent poor prognostic factors of patients with T4a stage gastric cancer.

**Conclusions:**

Preoperative serum tumor marker might be a candidate for the staging system in addition to conventional factors.

## Background

Gastric cancer was the fourth common cancer worldwide, and it was the second most common cause of death from cancer [1]. Although the survival of gastric cancer had been improved dramatically in some countries, especially in Japan, possibly due to early diagnosis following a great number of endoscopic examinations performed for gastric disorders, survival was still relatively low in North America and Western Europe even in cases treated by radical surgery, which raised the question whether the molecular pathology of gastric cancer was similar worldwide [2-4]. Therefore, a major impetus for the current study was the lack of previous studies on a large-scale Chinese population evaluating the expression of molecular prognostic markers in gastric cancer patients. Because of the variability of prognosis within a clinical or pathological stage of gastric cancer at presentation, which showed tumor stage could not provide complete information on the clinical behavior of gastric cancer, there has been a constant search for specific biological markers to identify subgroups of patients with more aggressive course of disease [5-7]. Some serum tumor markers including alpha-fetoprotein (AFP), carcinoembryonic antigen (CEA), carbohydrate antigen (CA) 19-9, CA50, and CA72-4 have been reported to be elevated in some patients with gastric cancer [8-10]. AFP, discovered about half a century ago by Abelev *et al.*, was a sensitive marker for diagnosis of hepatocellular cancer [11]. The serum level of AFP also increased in AFP-producing gastric cancer [12,13]. CEA, originally described in 1965 by Gold and Freedman, was routinely used as a serum marker for colorectal cancer [14]. CEA was a glycoprotein from the family of immunoglobulins whose function was to promote cellular binding. CA19-9, first described by Koprowski *et al.* in 1979 as a marker for colorectal cancer, had become the most important tumor marker for pancreatic adenocarcinoma [15]. CA19-9 was a high-molecular weight mucin that played a role in the adhesion of cancer cells to endothelial cells. CA50, initially screened out of colorectal cancer cell lines by Holmgren *et al.* in 1984, was a kind of glycolipid antigen that played an important part in growth and differentiation of cell [16]. CA72-4 was a complex glycoprotein that elevated in the serum of patients’ breast, pancreatic, ovarian, colon, and gastric cancers. CA72-4 was regarded as one of the most specific and sensitive markers for gastric cancers. However, it has not been routinely tested for gastric cancer patients in our hospital before 2005. At the present time, the value of these tumor markers in T4a stage gastric cancer was still elusive, which was responsible for more than 40% of gastric cancer and likely to present abnormal serum level of tumor markers. In this retrospective study, we evaluated the association between tumor markers and clinicopathological features and the prognostic value of tumor markers in T4a stage gastric cancer.

## Methods

### Patients

In total, 273 patients with histologically confirmed primary gastric adenocarcinoma underwent curative gastrectomy at the Department of Abdominal Surgery, Cancer Hospital, Fudan University between January 1996 and December 2005. Data were retrieved from their operative and pathological reports, and follow-up data were obtained by phone, outpatient clinical database, and letter. Informed consent was given to all participants. Ethical approval was given by Cancer Hospital. The study comprised 192 men and 81 women aged 22 to 78 years, mean age was 56 ± 12 years. There were 117 patients aged more than 59 years; the ratio of men to women was 192:81; 14 patients had a family history of gastric cancer; 198 patients liked to eat fried food, 50 patients liked to eat food rich in fat, and the other 25 patients had no special preference to fried food or fatty food; 64 cases had a history of smoking. Staging was performed according to the American Joint Committee on Cancer (AJCC) TNM Staging Classification for Carcinoma of the Stomach (Seventh Edition, 2010) [17]. All the patients belonged to T4a gastric cancer according to the AJCC/TNM Staging System. Gastrectomy was performed in accordance with the Japanese Classification of Gastric Carcinoma [18]. D2 gastrectomy, complete dissection of the first-tier and second-tier lymph nodes, was performed in all 273 patients. In each case, 15 or more lymph nodes were dissected according to the AJCC/TNM classification. A follow-up of all patients was carried out according to our standard protocol (every 3 months for at least 2 years, every 6 months for the next 3 years, and after 5 years every 12 months for life). The check-up items included physical examination, tumor-marker examination, ultrasound, chest radiography, computed tomographic scan, and endoscopic examination. The median follow-up time was 61.2 months for patients still alive at the time of analysis.

### Serum assays for AFP, CEA, CA19-9, and CA50

Blood samples were obtained from all patients in the morning during the week before surgery. The blood sample was centrifuged at 1000 g for 10 min to separate the plasma from the blood cells. AFP, CEA, CA19-9, and CA50 were assayed with magnetic particle enzyme immunoassay in UniCel^TM^ DxI 800 Access immunoassay system (Beckman Coulter Inc. Miami of U.S.A). The cutoff value for serum AFP, CEA, CA19-9, and CA50 were 10 μg/L, 10 μg/L, 37 U/mL, and 20 U/mL, respectively, according to the manufacturer’s instructions.

### Adjuvant chemotherapy

A total of 223 patients received adjuvant chemotherapy within 4 weeks after surgery. There were four kinds of chemotherapy regimens in our study: (1) oral administration of tegafur 600 mg per day for at least 1 year (*n* = 80); (2) oral administration of doxifluridine 1200 mg per day for at least 1 year (=65); (3) a combination of 5-fluorouracil, cisplatin, and mitomycin C, 500 mg/m^2^ 5-fluorouracil was administrated by intravenous infusion from days 1 to 5, 20 mg/m^2^ cisplatin intravenous from days 1 to 5, and 8 mg mitomycin C intravenous on day 1, then repeated every 21 days for at least six cycles (*n* = 43); and (4) combination of 5-fluorouracil and hydroxycamptothecine, intravenous administration of 500 mg/m^2^ 5-fluorouracil, and 8 mg/m^2^ hydroxycamptothecine from days 1 to 5, repeated every 21 days for at least six cycles (*n* = 43).

### Statistical methods

The association between tumor markers and clinicopathological factors was evaluated by Chi-square test. The 5-year survival rates were calculated by Kaplan-Meier method [19], and differences between survival curves were examined with log-rank test. The independent prognostic value of tumor markers and clinicopathological features was analyzed by Cox proportional hazards model [20]. Differences were considered statistically significant when the *P* value was < 0.05. Statistical analysis and graphics were performed with the SPSS 16.0 statistical package.

## Results

### Patients’ characteristics

Of the 273 reviewed patients, 90 patients had tumors located in the upper third of the stomach, 52 patients had tumors in the middle third, 124 patients had tumors in the lower third, and seven patients had tumors occupying two-thirds of the stomach or more. The distribution of postoperatively pathological stages of the patients was as follows: 49 patients belonged to IIB stage, 49 patients to IIIA stage, 52 patients to IIIB stage, and 123 patients to IIIC stage. Partial gastrectomy was performed in 193 patients, and total gastrectomy was performed in 80 patients.

### Positive rates of tumor markers

The preoperative serum positive rates of AFP, CEA, CA19-9, and CA50 were 5.9%, 16.1%, 32.6% and 29.7%, respectively. The serum value of AFP ranged from 0 to 3000 μg/L (mean 17.26 μg/L, and median 1.85 μg/L), CEA from 0 to 401 μg/L (mean 15.84 μg/L, and median 1.67 μg/L), CA19-9 from 1 to 1000 U/mL (mean 68.81 U/mL, and median 15.58 U/mL), CA50 from 0 to 549 U/mL (mean 37.02 U/mL, and median 12.00 U/mL).

### Correlation analysis

Patients with positive CEA, CA19-9, or CA50 showed a more advanced tumor stage than those with negative values (*P* =0.001, 0.000, and 0.000, respectively). Presence of occupying two-third of stomach or more was more frequent in those patients with positive CEA, CA19-9, or CA50 (*P* =0.014, 0.011, 0.004, respectively). The proportions of young patients were significantly higher in those with elevated serum CA19-9 (*P* = 0.047) and CA50 (*P* = 0.017) levels than those with normal levels. A statistically significant positive rate was found for CEA (*P* =0.029) level in males. Borrmann IV was more frequent in those with positive AFP (*P* =0.010). The patients with lymphovascular invasion more frequently showed higher values of CA19-9 (*P* =0.004). Patients with large-sized tumors were associated with significantly high positive rates of CA50 (*P* =0.014). The status of nervous invasion did not influence the positivity of the tumor markers (Table 1).

**Table 1:** Serum tumor markers and clinicopathologic factors of the patients

**Factors (*n*)**	**AFP(+)** **n (%)**	***P***	**CEA(+)** **n (%)**	***P***	**CA19-9(+)** **n (%)**	***P***	**CA50(+)** **n (%)**	***P***
*Sex*		0.408		0.029		0.213		0.242
Male (192)	13 (6.8)		37 (19.3)		67 (34.9)		61 (31.8)	
Female (81)	3 (3.7)		7 (8.6)		22 (27.2)		20 (24.7)	
*Age (years)*		0.053		0.779		0.047		0.017
≤40 (26)	4 (15.4)		3 (11.5)		13 (50.0)		13 (50.0)	
>40 (247)	12 (4.9)		41 (16.6)		76 (30.8)		68 (27.5)	
*Tumor site*		0.153		0.014		0.011		0.004
Upper (90)	5 (5.6)		16 (17.8)		31 (34.4)		31 (34.4)	
Middle (52)	3 (5.8)		5 (9.6)		19 (36.5)		15 (28.9)	
Lower (124)	6 (4.8)		19 (15.3)		33 (26.6)		29 (23.4)	
≥Two-third (7)	2 (28.6)		4 (57.1)		6 (85.7)		6 (85.7)	
*Tumor size*		0.063		0.812		0.102		0.014
≤6 (178)	7 (3.9)		28 (15.7)		52 (29.2)		44 (24.7)	
>6 (95)	9 (9.5)		16 (16.8)		37 (38.9)		37 (38.9)	
*Borrmann type*		0.010		0.365		0.086		0.066
I (15)	0 (0.0)		2 (13.3)		4 (26.7)		3 (20.0)	
II (8)	1 (12.5)		0 (0.0)		4 (50.0)		2 (25.0)	
III (228)	10 (4.4)		36 (15.8)		69 (30.3)		64 (28.1)	
IV (22)	5 (22.7)		6 (27.3)		12 (54.6)		12 (54.6)	
*N stage*		0.065		0.001		0.000		0.000
N0 (49)	0 (0.0)		3 (6.1)		7 (14.3)		6 (12.2)	
N1 (49)	1 (2.0)		6 (12.2)		14 (28.6)		14 (28.6)	
N2 (52)	4 (7.7)		3 (5.8)		10 (19.2)		9 (17.3)	
N3 (123)	11 (8.9)		32 (26.0)		58 (47.2)		52 (42.3)	
*Nervous invasion*		0.156		0.520		0.051		0.294
+ (197)	9 (4.6)		30 (15.2)		71 (36.0)		62 (31.5)	
- (76)	7 (9.2)		14 (18.4)		18 (23.7)		19 (25.0)	
*Lymphovascular invasion*		0.399		0.212		0.004		0.085
+ (196)	10 (5.1)		35 (17.9)		74 (37.8)		63 (32.7)	
- (77)	6 (7.8)		9 (11.7)		15 (19.5)		17 (22.1)	

Figures shown in parentheses are percentages.

### Univariate analysis

**Figure 1:**
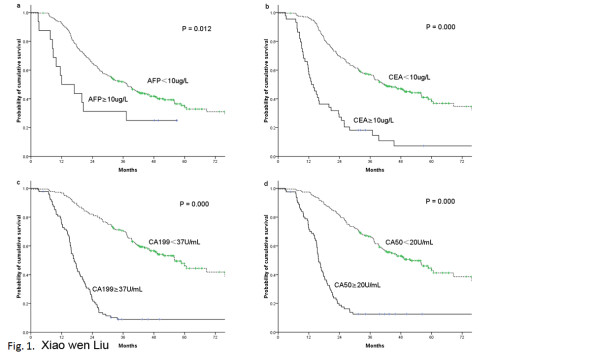
**Comparison of cumulative curves according to AFP level, CEA level, CA19-9 level, and CA50 level.** Patients with elevated serum AFP, CEA, CA19-9, or CA50 levels had a significantly worse prognosis than patients with normal levels of either marker (*P* =0.012, 0.000, 0.000, and 0.000, respectively).

The over-all 5-year survival rate was 38.1% for all 273 patients. The significant prognostic factors included: the serum level of AFP, CEA, CA19-9, and CA50 (Figure 1), age, tumor size, tumor site, Borrmann type, lymph node stage, nervous invasion, and lymphovascular invasion (Table 2). The 5-year survival was lower in patients with elevated AFP (*P* =0.012), CEA (*P* =0.000), CA19-9 (*P* =0.000), or CA50 (*P* =0.000) compared with those patients with normal levels of tumor markers. The 5-year survival was longer in patients with pN0 or pN1 than patients with pN2 or pN3 (*P* =0.000), in older patients (*P* =0.038), in patients with small size tumor (*P* =0.003), in patients with a presence occupying less than two-thirds of the stomach (*P* =0.008), in patients without Borrmann IV (*P* =0.017), in patients without nervous invasion (*P* =0.000) or lymphovascular invasion (*P* =0.000). The sex, adjuvant chemotherapy did not show any relationship with survival.

**Table 2:** Predictors of overall survival according to the univariate analysis

**Factors (*n*)**	**5-year survival (%)**	**Log-rank χ**^**2**^	***P***
*AFP*		6.287	0.012
≥10 (16)	25.0		
<10 (257)	38.9		
*CEA*		58.940	0.000
≥10 (44)	11.4		
<10 (229)	43.2		
*CA19-9*		131.705	0.000
≥37 (89)	10.1		
<37 (184)	51.6		
*CA50*		104.377	0.000
≥20 (81)	13.6		
<20 (192)	48.4		
*Sex*		0.001	0.978
Male (192)	38.5		
Female (81)	37.0		
*Age (years)*		4.300	0.038
≤40 (26)	23.1		
>40 (247)	39.7		
*Tumor site*		11.754	0.008
Upper (90)	34.4		
Middle (52)	34.6		
Lower (124)	43.5		
≥Two-third (7)	14.3		
*Tumor size*		8.687	0.003
≤6 (178)	42.7		
>6 (95)	29.5		
*Borrmann type*		10.158	0.017
I (15)	40.0		
II (8)	50.0		
III (228)	39.0		
IV (22)	22.7		
*N stage*		62.666	0.000
N0 (49)	67.3		
N1 (49)	53.1		
N2 (52)	44.2		
N3 (123)	17.9		
*Nervous invasion*		17.848	0.000
+ (197)	29.9		
-(76)	59.2		
*Lymphovascular invasion*		17.242	0.000
+ (196)	30.6		
-(77)	57.1		
*Adjuvant chemotherapy*		1.443	0.230
Yes (223)	39.0		
No (50)	34.0		

### Multivariate analysis

Multivariate survival analysis was performed by evaluating all significant prognostic factors from univariate analysis to determine the independent prognostic factors for T4a stage gastric cancer. Multivariate analysis using Cox proportional hazards model showed that tumor markers, including CEA, CA19-9, and CA50, were independent prognostic factors, as tumor size, lymph node stage, and nervous invasion. According to the relative risk, these independent prognostic factors were ranked as CEA, CA19-9, nervous invasion, CA50, pN stage, and tumor size by descending (Table 3).

**Table 3:** Independent prognostic factors at multivariate analysis by Cox Model

**Factors**	**Hazard ratio**	**95% CI**	***P***
CEA	2.809	1.823–4.327	0.000
CA19-9	2.740	1.620–4.635	0.000
CA50	2.091	1.236–3.538	0.006
Tumor size	1.595	1.147–2.219	0.006
pN stage	1.624	1.378–1.914	0.000
Nervous invasion	2.510	1.456–4.325	0.001

### Comparison of survival according to CEA, CA19-9, and CA50

**Figure 2:**
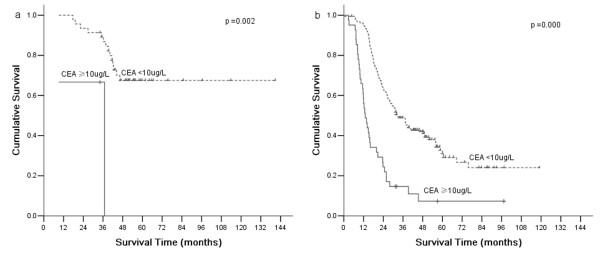
**Comparison of survival according to stages II and III.** A significant difference in survival of patients with stages II and III was observed between positive CEA and negative CEA (*P* =0.002 and 0.000, respectively).

**Figure 3:**
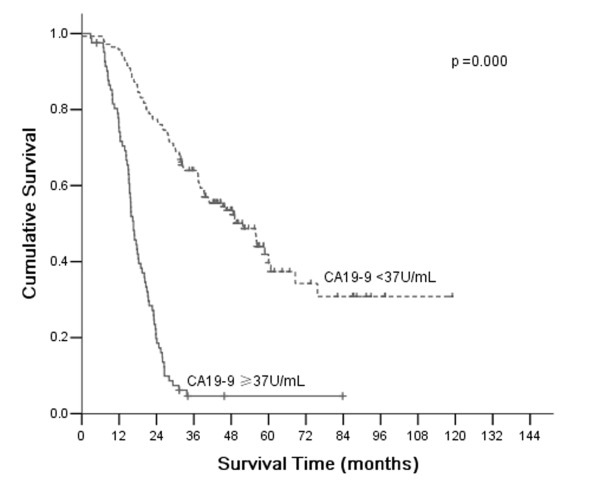
**Comparison of survival according to stages II and III.** A significant difference in survival of patients with stage III was observed between positive CA19-9 and negative CA19-9 (*P* = 0.000).

**Figure 4:**
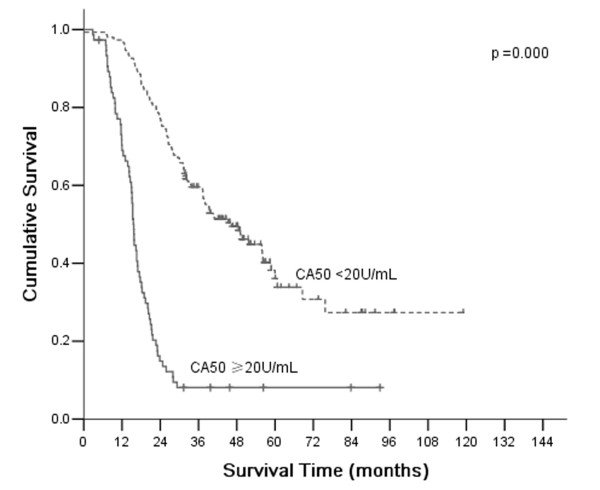
**Comparison of survival according to stages II and III.** A significant difference in survival of patients with stage III was observed between positive CA50 and negative CA50 (*P* =0.000).

According to AJCC/TNM Staging System, T4a stage gastric cancer was divided into four stages: IIB, IIIA, IIIB, and IIIC. Based on CEA, CA19-9, and CA50, which are all independent prognostic factors, stages II and III were divided into CEA (+) and CEA (-), CA19-9 (+) and CA19-9 (-), CA50 (+) and CA50 (-), respectively. There were significant differences of overall 5-year survival between CEA (+) and CEA (-) according to stage II and III (*P* =0.002 and 0.000, respectively; Figure 2). There were significant differences of overall 5-year survival between CA19-9 (+) and CA19-9 (-) according to stage III (*P* =0.000; Figure 3). There were significant differences of overall 5-year survival between CA50 (+) and CA50 (-) according to stage III (*P* =0.000; Figure 4).

## Discussion

The main findings of this study were: (1) tumor markers including CEA, CA19-9, and CA50 were independent prognostic factors for T4a stage gastric cancer; and (2) there were significant differences of overall 5-year survival rate between CEA (+) and CEA (-) according to stages II and III; between CA19-9 (+) and CA19-9 (-) according to stage III; between CA50 (+) and CA50 (-) according to stage III.

Various tumor markers have been identified since Gold and Freedman first reported the discovery of carcinoembryonic antigen (CEA) in 1965 [14]. These tumor markers have been studied primarily for applicability and feasibility in terms of tumor early detection. Among these tumors, AFP, CEA, CA19-9, CA50, and CA72-4 were considered as relatively specific markers for gastric cancers. In particular, CA 72-4 was regarded as one of the most specific and sensitive markers for gastric cancers. However, as a retrospective study, we could only evaluate the prognostic value of AFP, CEA, CA19-9, and CA50 for gastric cancers, which were evaluated in our institute. A few of studies have investigated the value of various tumor markers, including CEA, CA19-9, and CA50 in gastric cancer [21-23]. Some reports showed that one or more tumor markers could predict specific clinical outcomes such as prognosis, response to treatment, and recurrence, although results were not consistent. Furthermore, these studies have been counteracted by several methodologic flaws: (1) the inclusion of patients with various tumor stages raging from localized to metastatic; and (2) a heterogeneous treatment regimen. So it is necessary to clarify the value of tumor markers in homogeneous group of patients with gastric cancer.

We only evaluated patients with T4a N0-3 M0 gastric cancer, who received D2 gastrectomy. The reasons for including only T4a gastric cancers as follows: (1) There have been some studies on the prognostic impact of tumor markers in gastric cancer, but rarely have the previous studies evaluated the prognostic impact of tumor markers when specific depth of invasion was involved, especially in the T4a classification which was responsible for more than 40% of gastric cancers. Therefore, the precise determination of the prognostic value of tumor markers in T4a gastric cancer has substantial clinical importance. (2) Since some previous studies showed an association between the depth of tumor invasion and serum tumor markers levels [24,25], it was difficult to identify the important prognostic factors as a result of interrelated factors. In each case, 15 or more lymph nodes were dissected according the AJCC/TNM classification. In this study, the positive rate of patients with elevated serum AFP levels was 5.9%, which was similar to those reported by other investigators [12,24,26]. The positive rate of CEA and CA19-9 were 16.1% and 32.6%, respectively, which are lower than that of other studies [27,28]. The corresponding proportion of patients with elevated serum CA50 levels was 29.7%. The positive rates of tumor markers are thought to be influenced by tumor progression at the time of presentation. When the serum positive rates of AFP, CEA, CA19-9, and CA50 were evaluated according to TNM stage grouping, it showed that the positive rates increased gradually with stage.

We compared other clinicopathological factors between patients with elevated tumor markers and those with normal levels of serum tumor markers. The proportion of patients with elevated serum AFP was significantly higher in those with Bormmann IV. The elevated serum CEA was associated with gender, tumor site, and pN stage. The elevated serum CA19-9 was associated with age, tumor site, lymphovascular invasion, and pN stage. CA50 positivity was significantly associated with age, tumor size, tumor site, and pN stage. We found that these tumor markers were associated with pN stage. This finding indicated that the positive rates of tumor markers increased as the tumor progressed. This was agreement with previous studies of AFP, CEA, and CA19-9 [25,26]. Additionally, we found that the positive rate of CEA was higher in males than in females, which was not consistent with previous some studies [26,29]. It was possible that the limited samples in these studies contributed to the negative correlation between CEA and gender. This result indicated that the serum CEA should be investigated in patients with gastric cancer, especially for male patients.

According to univariate analysis, our results showed that there was a significant difference in 5-year overall survival in terms of tumor markers and distinct clinicopathologic factors, which included age, tumor size, tumor site, Borrmann type, lymph node stage, nervous invasion, and lymphovascular invasion. We found that the serum levels of AFP, CEA, CA19-9, and CA50 were significantly correlated with survival rate in patients with T4a stage gastric cancer, which was in agreement with previous studies of AFP, CEA, and CA19-9 [25,26,29]. These correlations indicated that patients with positive values of tumor markers have worse prognosis, which in turn may be due to the predominant proportion of advanced gastric cancer in this cohort of patients.

Multivariate analysis using Cox proportional hazards model showed that tumor markers including CEA, CA19-9, CA50, were independent prognostic factors, as tumor size, lymph node stage, and nervous invasion. The Cox proportional hazards regression analysis showed that patients with elevated levels of CEA, CA19-9, and CA50 had a higher risk of death than patients with low levels of these markers. Except CA50, the prognostic value of CEA and CA19-9 in gastric cancer had been widely studied. Tocchi *et al*. [30] found that CEA and CA19-9 provided independent predictive value in gastric cancer patients, but the other studies did not show consistent result [26,29]. This was likely due to the heterogeneity of patients included in these studies, including those with localized disease who undergo gastrectomy and those with locally advanced and disseminated disease who may not undergo resection.

To evaluate whether serum CEA, CA19-9, and CA50 could provide additional prognostic information on the basis of AJCC/TNM stage system, we compared cumulative survival curves according to CEA, CA19-9, and CA50. The results showed that there were significant differences between patients with elevated levels and those with normal serum CEA, CA19-9, and CA50 levels at stage III. These findings indicated that serum CEA, CA19-9, and CA50 levels could provide additional prognostic information in part of the patients with T4a stage gastric cancer. Similar results in patients with gastric cancer have been reported by others. A previous study reported that the survival rate of gastric cancer patients at stages I, II, and III with elevated serum CEA levels was significantly poorer than that of patients with normal levels [31]. Another study reported that there were significant differences between patients with elevated CA19-9 levels and those with normal levels at stage I [23]. The reason that preoperative levels of tumor markers could influence long-survival of T4a gastric cancer was still unclear; it was possible that a number of biological factors are involved. CEA and CA19-9 belonged to intercellular adhesion molecules, so cells expressing these glycoproteins may have a greater invasive potential [8]. CA50 acted as a kind of glycolipid antigen that played a role in growth and differentiation of cell, suggesting that cells expressing this antigen would possess increased proliferating activity [16].

Some limitations of this study should be acknowledged. Firstly, we only evaluated patients with T4a N0-3 M0 gastric cancer. Although this is a design of the study, only inclusion of T4a stage gastric cancers would limit the application of the results to the early stage or more unfavorable moderate stage gastric cancer. Secondly, CA72-4 was not routinely tested for gastric cancer before 2005 in our hospital, therefore its predicting value for gastric cancer was not known. Thirdly, as a result of uncompleted data about recurrence, it was unable to evaluate the correlation between tumor markers and recurrence. Fourthly, we could not determine the prognostic value of peritoneal cytology in our study, because we did not perform peritoneal cytology in the management of gastric cancer. Therefore, this may have influenced the survival data. In addition, some other molecular markers such as cell-free DNA (cfDNA) and miRNA should be investigated in future. Previous studies have demonstrated an increase of circulating cfDNA in different types of cancer [32]. MicroRNA (miRNA) played an important role in regulating gene express. Chen *et al.*[33] found that expression pattern of serum miRNA was altered in reflection of various disease.

## Conclusions

In conclusion, tumor marker, which can be easily measured before surgery, is a simple and reliable prognostic factor in T4a stage gastric cancer. Therefore it might be a candidate for the staging system in addition to conventional factors. However, as a retrospective study, our data could not allow us to directly address some issues. Large-scale, prospective studies, which combined tumor markers and molecular markers like cfDNA and miRNA, are needed to answer above mentioned questions in future.

## Competing interests

There is no any conflict of interest about the study.

## Author contributions

Conceived and designed the experiments: XWL HC YNW. Performed the experiments: XWL. Analyzed data: XWL YNW. Wrote the paper: XWL. All authors read and approved the final manuscript.

## Synopsis

Tumor marker, which can be easily measured before surgery, is a simple and reliable prognostic factor in T4a stage gastric cancer. Therefore it might be a candidate for the staging system in addition to conventional factors.
